# Clinical Validation of Plasma Metabolite Markers for Early Lung Cancer Detection

**DOI:** 10.3390/ijms26104519

**Published:** 2025-05-09

**Authors:** Lun Zhang, Jiamin Zheng, Rashid A. Bux, Jean-François Haince, Claudia Torres-Calzada, Rupasri Mandal, Andrew Maksymiuk, Guoyu Huang, Paramjit S. Tappia, Philippe Joubert, Christian D. Rolfo, David S. Wishart

**Affiliations:** 1 The Metabolomics Innovation Centre, University of Alberta, Edmonton, AB T6G 2E8, Canada; lun2@ualberta.ca (L.Z.); jiamin3@ualberta.ca (J.Z.); rmandal@ualberta.ca (R.M.); 2 BioMark Diagnostics Inc., Richmond, BC V6X 2W2, Canada; rahmed@biomarkdiagnostics.com (R.A.B.); jhaince@biomarkdiagnostics.com (J.-F.H.); ghuang@biomarkdiagnostics.com (G.H.); 3 Department of Biological Sciences, University of Alberta, Edmonton, AB T6G 2E8, Canada; ctorresc@ualberta.ca; 4 Cancer Care Manitoba, Winnipeg, MB R3E 0V9, Canada; amaksymiuk@cancercare.mb.ca; 5 Department of Internal Medicine, Rady Faculty of Health Sciences, University of Manitoba, Winnipeg, MB R3A 1R9, Canada; 6 Asper Clinical Research Institute, St. Boniface Hospital, Winnipeg, MB R2H 2A6, Canada; ptappia@sbrc.ca; 7 Institut Universitaire de Cardiologie et de Pneumologie de Québec-Université Laval, Quebec, QC G1V 4G5, Canada; philippe.joubert@criucpq.ulaval.ca; 8 Department of Internal Medicine, Division of Medical Oncology, The Arthur G. James Comprehensive Cancer Center, 460 W 12th Avenue, Columbus, OH 43210, USA; christian.rolfo@osumc.edu; 9 Department of Computing Science, University of Alberta, Edmonton, AB T6G 2E8, Canada; 10 Department of Laboratory Medicine and Pathology, University of Alberta, Edmonton, AB T6G 2B7, Canada; 11 Faculty of Pharmacy and Pharmaceutical Sciences, University of Alberta, Edmonton, AB T6G 2H7, Canada

**Keywords:** metabolite markers, early detection, non-small cell lung cancer

## Abstract

Early detection of lung cancer significantly improves survival, yet current screening methods have limitations. This study aimed to identify a robust panel of plasma metabolites for early-stage non-small cell lung cancer (NSCLC) diagnosis using a large, clinically diverse patient cohort. A total of 680 archived plasma samples from biopsy-confirmed NSCLC patients and controls (including healthy individuals and patients with non-cancerous lung diseases) were analyzed using targeted, quantitative mass spectrometry-based metabolomics and used as the discovery cohort. An independent set of 216 plasma samples served as the validation cohort. Logistic regression (LR) models developed from the discovery set using ten metabolites achieved area under the receiver-operating characteristic curve (AUROC) values of 93.63%, 93.74%, and 93.91% for distinguishing all-stage, stage I–II, and stage I NSCLC patients from controls, respectively. Incorporating smoking history further improved model performance. The validation cohort confirmed the model’s robustness, demonstrating high sensitivity and specificity for early-stage detection. These results support the potential of metabolomic biomarkers as a minimally invasive, accurate tool for early NSCLC diagnosis. This approach may complement current screening methods, enabling earlier intervention and improved patient outcomes. Further studies are warranted to validate these findings in more diverse populations and real-world clinical settings.

## 1. Introduction

Lung cancer ranks among the primary causes of cancer-related fatalities worldwide. It is a complex disease that arises from the uncontrolled growth of abnormal cells in lung tissue. There are two main types of lung cancer: non-small cell lung cancer (NSCLC) and small cell lung cancer (SCLC). NSCLC accounts for approximately 85% of all lung cancer cases and is further classified into three subtypes: adenocarcinoma, squamous cell carcinoma, and large cell carcinoma [1]. According to the American Cancer Society and the Canadian Center for Applied Research in Cancer Control, lung cancer is among the most commonly diagnosed cancers in Canada and the US, as well as the leading cause of cancer-related deaths in both countries [2,3]. The diagnosis and treatment of lung cancer are challenging due to its diverse subtypes, varying stages, spatial-temporal heterogeneity, and complex biological mechanisms.

Early detection of lung cancer is crucial for improving patient outcomes, as early diagnosis is associated with higher survival rates. Patients with stage IA lung cancer have a high 5-year survival rate exceeding 75%, but as cancer progresses, the long-term survival rate dramatically decreases [4]. Over the years, various methods have been developed for detecting lung cancer, ranging from traditional imaging techniques to cutting-edge molecular tests. The most common methods to detect lung cancer include imaging techniques such as chest X-rays, computed tomography (CT) scans, and positron emission tomography (PET) scans. These methods can help detect lung nodules or tumors and determine their size and location. However, imaging tests may not always provide a definitive diagnosis, and further testing such as lung biopsies may be required. Molecular tests such as liquid biopsies, which detect circulating tumor DNA (ctDNA) or other biomarkers in blood samples, and genetic testing of lung cancer tissue, can also aid in the diagnosis and management of lung cancer [5]. The application of low-dose computed tomography (LDCT) in the context of lung cancer screening programs provides the possibility of detecting lung cancer at earlier, more operable stages in high-risk populations. However, LDCT by itself is a limited approach, with lower sensitivity and specificity compared with regular chest CT scan [6,7].

In recent years, metabolomics has emerged as a promising tool in the field of cancer research, including lung cancer. Metabolomics is the comprehensive study of metabolites, which are small molecules produced by cellular processes. Metabolomics not only provides a snapshot of the metabolic state of a cell or organism [8], but it can also be used to identify biomarkers for the early diagnosis of lung cancer, reveal the pathogenesis of the disease, and provide insights into potential therapeutic targets [9,10,11,12]. By analyzing the metabolic profiles of biological samples, such as blood, plasma, or urine, metabolomics can detect changes in metabolite levels that are associated with lung cancer [13]. This approach has the potential to improve the accuracy and sensitivity of lung cancer detection, leading to the development of more effective diagnostic strategies. In this context, metabolomics holds great promise for improving the detection, diagnosis, and treatment of lung cancer. However, further research is needed to validate metabolomic biomarkers and identify their clinical utility in the management of this devastating disease.

The purpose of this study is to build upon our previous research on lung cancer [14] and to identify a more robust panel of plasma metabolites for early-stage lung cancer diagnosis by using a much larger and more clinically complex patient cohort. While earlier studies [13,14] have demonstrated the promise of metabolomics in distinguishing lung cancer patients from healthy controls, further validation and identification of a more reliable set of biomarkers in a control cohort that includes individuals with other non-cancerous lung diseases, is essential. To achieve this, we employed the same quantitative MS-based metabolomics assay used in [14] to analyze a significantly larger set of plasma samples from both lung cancer patients and non-cancer controls (many with other lung diseases). The resulting high-performing biomarker panel was then validated on a slightly smaller cohort with a similarly complex patient structure and found to exhibit essentially the same high diagnostic performance. This work has the potential to improve the clinical utility of metabolomics in the early detection and diagnosis of lung cancer, ultimately leading to better patient outcomes.

## 2. Results

### 2.1. Clinical Cohorts

A summary of the clinical variables for both the discovery set and the validation set is listed in Table 1. The results from a comparison of the different sets can be found in Table S1. Comparisons of weight and BMI between each cancer group and the control group revealed no statistically significant differences. The χ^2^ test results showed that the cancer groups and the controls were well matched in terms of sex. However, the Mann–Whitney rank sum tests highlighted a significant age difference between the controls and each cancer group. Despite the minor fold-change in age between the controls and each cancer group (for instance, the age fold-change in Stage I and Stage II versus controls is 1.06 and 1.08, respectively), the significance impact of age was assessed by including it as a covariate in the subsequent modeling phase.

Furthermore, it is important to note that there are differences in the smoking status between the cancer groups and the control group, as outlined in Table 1. The correlation between smoking history and lung cancer is a topic that has been extensively researched and is widely recognized. In our dataset, a very significant association between smoking history and the incidence of lung cancer was observed. For instance, when considering all cancer patients as a single group, the *p*-value of the χ^2^ test was 2.2 × 10^−16^. Moreover, both sets exhibited notably higher medians of smoking amount (measured in packs × years) in the cancer groups compared to the control groups. As a result, the smoking status was included as a cofactor in the subsequent modeling due to its relevance and potential influence.

### 2.2. Univariate and Multivariate Statistical Analysis

**Figure 1:**
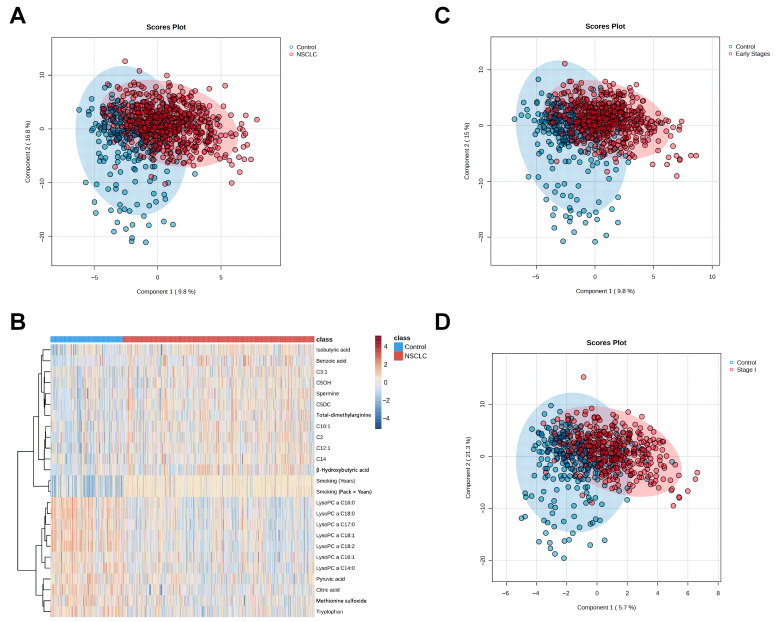
Metabolomic profiles of lung cancer patients and controls are significantly different. (**A**) PLS-DA 2D-scores plot of all-stages lung cancer patients vs. controls. (**B**) A hierarchical clustering heat map of the metabolites measured in the plasma of lung cancer patients and healthy controls. Only the top 25 metabolites were shown. (**C**,**D**) PLS-DA 2D-scores plot of early stages (stages I + II) lung cancer patients (**C**), and Stage I lung cancer patients (**D**) vs. controls.

In our previous study [14], a significant distinction between the metabolomic profiles of patients with NSCLC and healthy individuals was discovered. In this study, a similar set of analyses was conducted initially for all NSCLC patients and controls. All metabolic features in the dataset were processed as outlined in the Materials and Methods Section. These analyses aimed to explore the alterations in the metabolomic profiles of the patients and to identify potential biomarkers associated with the disease. The PLS-DA analysis revealed distinct metabolomic profiles between patients with NSCLC and control individuals (Figure 1A). The permutation test confirmed that the class discrimination is significant (Figure S1). In the exploratory receiver-operating characteristic (ROC) analysis, based on random forest, the area under the ROC curve (AUROC) of different models, each with different number of metabolite features, ranged from 0.80 to 0.90 (Figure S2A). These results suggest that metabolic features may effectively discriminate NSCLC patients from controls. The heatmap showed that patients with NSCLC had higher plasma concentrations of acyl-carnitines and beta-hydroxybutyric acid, but lower levels of lysophosphatidylcholines (lysoPCs), citric acid, pyruvic acid, and tryptophan, compared to the controls (Figure 1B).

The Mann–Whitney rank sum tests showed that 41 out of the 138 quantitatively measured metabolites displayed significant differences between the patients with NSCLC and the controls. Both univariate analyses (using the Mann–Whitney rank sum tests) and multivariate analyses (using the PLS-DA and the random forest ROC analysis) consistently identified the same metabolites with relevant alterations. The metabolites that increased most notably were β-hydroxybutyric acid, citrulline, carnitines (carnitine and acetyl-carnitine), and succinic acid. Conversely, the metabolites that decreased most significantly included citric acid, tryptophan, various lysoPCs (lysoPC a C18:2, lysoPC a C18:0, and lysoPC a C16:0), and PC ae C40:6 (Figure S2B and Table S2).

The earlier a set of biomarkers can diagnose a condition, the more valuable it becomes. Therefore, we sequentially investigated the metabolomic profiles of patients at the early stages (Stage I + II; n = 416) and at the earliest stage (Stage I; n = 275), following the same analysis workflow as described above. The PLS-DA results demonstrated a clear separation between the controls and the patients at different disease stages (Figure 1C,D). The permutation test confirmed that the observed PLS-DA results were not due to chance (Figures S3 and S5).

The AUROC of different random forest-based models, with varying numbers of metabolite features, ranged from 0.81 to 0.90 (Figure S4A) for the early-stage patients, and from 0.82 to 0.91 (Figure S6A), for the stage I patients. For both the early-stage patients (Stage I + II) and the Stage I patients alone, a similar combination of biomarkers was suggested by the PLS-DA (Figures S4B and S6B), the random forest-based ROC model (Figures S4C and S6C), and the Mann–Whitney rank sum tests (Tables S3 and S4). Considering that approximately 90% of the enrolled NSCLC patients were at Stages I and II, these results suggest that patients with NSCLC at Stage I and Stage II shared a similar metabolomics profile. In fact, the PLS-DA was unable to distinguish patients with NSCLC at different stages.

### 2.3. Logistic Regression Modeling

**Figure 2:**
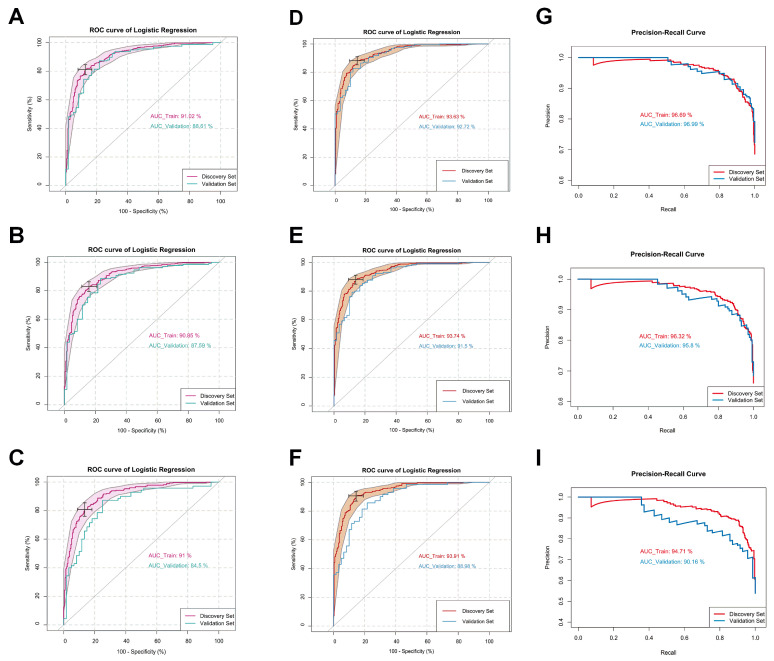
Logistic regression modeling can effectively discriminate lung cancer patients from controls: (**A**–**C**) ROC curves generated by the logistic regression models using metabolite features only for lung cancer patients at all stages (**A**), early stages (stage I + stage II) (**B**), and stage I (**C**). ROC curves and their 95% CI on the discovery set are shown in magenta. ROC curves obtained from the validation set are colored in cyan. (**D**–**F**) ROC curves generated by the logistic regression models using both metabolite features and smoking factor for lung cancer patients at all stages (**A**), early stages (stage I + stage II) (**C**), and stage I (**E**). ROC curves and their 95% CI on the discovery set are shown in red. ROC curves obtained from the validation set are colored in blue. (**G**–**I**) Precision–recall curves of the logistic regression models for lung cancer patients at all stages (**B**), early stages (stage I + stage II) (**D**), and stage I (**F**). Curves of the discovery set and the validation set are colored in red and blue, respectively.

Logistic regression (LR) was carried out to develop a diagnostic model of NSCLC with potential clinical utility. Initially, attention was focused solely on the metabolite features. It was suggested by the previous random forest-based ROC model exploration that high discriminative power (AUC > 85%) could be achieved by introducing no more than 15 metabolomic biomarkers (Figure S2A). Multiple LR models for all stages of NSCLC, early stages, and Stage I were built and optimized using the discovery set. A combination of 10 metabolites out of an initial set of 138 was consistently identified throughout the modeling process that yielded consistent results in terms of diagnostic performance across different cancer stages. These metabolites included citric acid, tryptophan, lysoPC a C18:2, lysoPC a C20:3, carnitine, glutamine, citrulline, succinic acid, PC aa C38:0, and PC ae C40:6. Nearly identical performance in both the discovery and the validation sets was achieved by the diagnostic model. When all patients with NSCLC were included, the AUROC values for the discovery set and the validation set were 91% and 89%, respectively. When applied to early-stage patients and Stage I-only patients, the discovery-AUROC/validation-AUROC values were 91%/88% and 91%/85%, respectively (Figure 2A–C).

As previously noted, an imbalance was observed in age and smoking amount between the NSCLC and the control groups. The significance of these potential confounders was determined by examining their influence during the modeling process. When age alone was used to build the logistic regression model, an AUROC value of 57% was achieved, with a corresponding *p*-value of 0.61. When age was added to the previously described LR model, a *p*-value of 0.33 was obtained and the AUROC value was only increased by 0.07%. These findings suggest that age does not play a significant role in discriminating between NSCLC patients and controls. In contrast, when only the smoking amount (packs × years) was considered, an AUROC value of 82% was achieved by the logistic regression model. The *p*-value associated with the smoking amount in the model was found to be less than 2.0 × 10^−16^, indicating its predictive power. Based on these findings, the decision was made to use the smoking amount (packs × years) as the clinical factor for subsequent modeling.

After the smoking amount was included in the LR model, AUROCs of 94% and 93% were achieved using the modified LR model for the discovery and validation cohorts, respectively (Figure 2D). The Youden index of the discovery AUROC curve achieved 85% sensitivity and 88% specificity. AUROCs of 97% and 97% were achieved by the precision–recall curve of the model for the discovery and validation cohorts, respectively (Figure 2G). When the smoking amount was included, the previously reported discovery-AUROC/validation-AUROC values were increased to 94%/92% and 94%/89%, for the early-stages patients (Stage I and II) and the Stage I-only patients, respectively (Figure 2E,F). The sensitivity and specificity of the LR model for the early-stages patients were 88% and 81%, respectively (Youden Index). For the Stage I patients, the sensitivity and specificity of 91% and 80%, respectively, were reached by the LR model (Youden Index). As shown in Figure 2H, the area under the precision–recall curves of the early-stage patients’ LR model were 96% for both the discovery set and the validation set. For the Stage I-only patients, 95% and 90% of the precision–recall curve AUROC values were achieved by the LR model, for the discovery set and the validation set, respectively (Figure 2I). Other details of the three models described above are listed in Table 2, Table 3 and Table 4.

Due to the imbalance in smoking status between cases and controls, we evaluated whether smoking status acted as a confounding factor in the predictive models. LR models were constructed using the 10-metabolite panel to differentiate smokers from non-smokers within the discovery set, where non-smokers were defined as individuals who had never smoked. As shown in Figure S7, the AUROCs of the models for all stages, early-stage, and stage I-only NSCLC patients were 76.82%, 84.47%, and 78.53%, respectively. These values are notably lower than the AUROCs achieved (which were all above 90%) when discriminating NSCLC patients from controls. This strongly suggests that the identified metabolite features were not primarily driven by smoking status. In addition, we assessed the correlations between smoking amount (pack × year) and the metabolite features. As summarized in Table S5, smoking amount exhibited slight negative correlations with all 10 metabolites. Furthermore, in the LR models for NSCLC discrimination, only 5 out of the 10 metabolites displayed odds ratios with trends opposite to those observed for smoking amount (Table 2, Table 3 and Table 4). Collectively, these results indicate that the identified metabolite panel reflects metabolic alterations associated with NSCLC, rather than merely capturing changes related to smoking.

## 3. Discussion

In both our previous and current studies, we applied quantitative LC-MS/MS-based metabolomics techniques to gain valuable insights into the metabolic alterations associated with NSCLC. The use of absolute or semi-quantitative data in our statistical methods and modeling allowed for a more precise analyses and achieve more consistent outcomes regarding alterations in the metabolomic profiles of individuals with NSCLC. Previously, we demonstrated that high-performance LR models could distinguish NSCLC patients from healthy individuals using the blood levels of β-hydroxybutyric acid, lysoPC 20:3, PC ae C40:6, citric acid, and fumaric acid. In this study, we applied the same quantitative techniques to a much larger sample from the same population to validate our previous findings. When we built the LR model using only the five previously published metabolites and the smoking frequency (measured as the amount of pack cigarettes smoked per year), the AUROC of the discovery set reached 87%. This result indicates that our previous study, despite its smaller sample size, correctly identified metabolomic differences in early-stage NSCLC patients compared to the non-cancer population.

To achieve a model with superior performance that fits both the discovery cohort and validation cohort, we conducted multiple statistical analyses and LR modeling. Ultimately, we selected ten metabolites to build a high-performance model for diagnosing early-stage NSCLC. β-hydroxybutyric acid and fumaric acid were excluded from the new model, while lysoPC 20:3, PC ae C40:6, and citric acid remained as significant diagnostic components. Of the ten metabolites selected for the model, two were from both the lysoPC and PC families. Decreased levels of polyunsaturated PCs have been previously reported in lung cancer [15], and altered lysoPC levels have also been documented in various cancer studies [16,17]. Although no definitive conclusions have been drawn regarding the relationship between lung cancer and these two lipid families, the consistent changes observed for lysoPC a C20:3, lysoPC a C18:2, PC ae 40:6, and PC aa C38:0 in both the discovery and validation sets strongly suggests that changes in these lipids families may be a characteristic of the blood metabolome in early-stage NSCLC patients.

In addition to these four lipids, the remaining six small-molecule metabolites included in the model have been widely associated with tumors [18,19,20,21,22,23,24,25,26,27,28]. Alterations in the concentrations of these metabolites in the blood of NSCLC patients may be related to the metabolic reprogramming of tumor tissues. We also examined the distribution of clinical variables such as age, smoking history, sex, race, height, weight, and BMI, among NSCLC patients and controls. Of the collected samples, only age and smoking history showed significant differences between NSCLC patients and controls. We then further explored the role of age and smoking history on the ability to discriminate NSCLC patients from controls. While the inclusion of age did not significantly affect the model’s performance, the addition of smoking history did improve it. Thus, the final model used metabolites and smoking frequency as biomarkers to differentiate between NSCLC and control groups.

This study minimized the influence of clinical variables on the metabolomics profiles, as the samples were balanced across groups for sex, race, and BMI. Although age and BMI did not significantly enhance the model’s performance, other studies have shown a significant association between these factors and the risk of NSCLC [29,30,31]. Future follow-up studies in different cohorts are necessary to explore the potential of incorporating BMI and age into the modeling to improve diagnostic accuracy. Additionally, lifestyle and environmental exposure factors are closely related to the risk of NSCLC. In subsequent studies, it will be important to consider how to integrate these factors into the diagnostic model to further improve its performance in real-world situations.

In this study, the control group included patients with non-cancerous lung diseases such as COPD, asthma, benign lung tumors, and bronchitis. These patients may exhibit symptoms similar to early-stage lung cancer, potentially complicating the clinical diagnosis of lung cancer. Despite this, our diagnostic model remained robust, highlighting its potential applicability in real-world lung-cancer-screening scenarios. Our findings also indicate significant differences in the metabolomic profiles between NSCLC and non-cancerous lung diseases. It is worth noting that patients with non-cancerous lung diseases accounted for 63% of the subjects enrolled in the control group. To ensure the transferability of our findings, future studies should validate our results using samples that reflect the distribution of patients with non-cancerous lung diseases in the target population of lung cancer screening programs.

All patient samples in this study were collected prior to any treatment, including surgical resection. Therefore, we do not currently have sufficient evidence to determine whether the levels of the identified metabolic biomarkers change following tumor removal. To address this issue, longitudinal studies incorporating post-resection sampling will be necessary to evaluate the temporal dynamics of these metabolites and to assess their potential utility in monitoring treatment response or disease recurrence.

The metabolomics-based model developed in this study holds significant clinical potential for early detection of NSCLC, especially when compared to currently available methods such as LDCT and liquid biopsy techniques like ctDNA assays. LDCT, while widely used for lung cancer screening, presents several limitations, including low specificity (61–73%), which often results in false positives and unnecessary follow-up procedures [6,7,32,33]. Our metabolomic model, by comparison, demonstrated much higher AUROC (above 90%) for early-stage NSCLC, suggesting improved specificity and fewer false positives. This makes it a strong candidate for its integration into existing screening programs, possibly reducing the burden of unnecessary diagnostic interventions.

On the other hand, ctDNA assays, while offering a non-invasive alternative, struggle with low sensitivity (50–70%) in detecting early-stage lung cancer. In contrast, our model’s ability to detect metabolic changes associated with early-stage NSCLC makes it a more sensitive and robust method for early detection. By focusing on the metabolic reprograming characteristics of NSCLC, our approach provides an advantage over ctDNA, which may need higher levels of circulating tumor material (found in later disease stages) for accurate detection [34,35,36].

Overall, the integration of our metabolomics-based method with traditional techniques could improve the accuracy and efficiency of lung-cancer-screening programs. The relatively low cost, minimal invasiveness, and high sensitivity of our method make it an appealing addition to clinical workflows, particularly for high-risk populations.

## 4. Materials and Methods

### 4.1. Regulatory and Institutional Review Board Approvals

Ethics approval was obtained from the University of Manitoba Health Research Ethics Board (Ethics File#: H2012:334) prior to the implementation of the study. Research ethics approval was also obtained from the University of Alberta (Study ID: Pro00093715) to conduct the metabolomic studies in Edmonton.

### 4.2. Study Population and Sample Collection

All plasma samples were obtained from the IUCPQ (Institut Universitaire de Cardiologie et de Pneumologie de Quebec) Biobank-Respiratory Healthy Research Network. Frozen (−80 °C) plasma aliquots ranging from 200 to 400 µL were shipped to The Metabolomics Innovation Center (TMIC) at the University of Alberta, Canada, for quantitative metabolomic analysis. The 680 archived plasma samples that were used as the discovery cohort in this study were collected from 466 patients with biopsy-proven and biopsy-graded lung cancer and 214 controls. The 216 plasma samples that were used as the validation set were collected from 156 patients with biopsy-proven and biopsy-graded NSCLC and 60 controls [14]. The cancer samples had comprehensive data, including demographics, body mass index (BMI), smoking status, overall survival, morbidities, pathology, etc. The control samples had data on demographics, BMI, and medical condition history. Patients and controls with a history of any liver or kidney disease, and any previous treatment with anti-neoplastic drugs were excluded from this cohort. In addition to healthy individuals, the control groups also included patients with various pulmonary conditions such as asthma, chronic obstructive pulmonary disease (COPD), bronchiectasis, hamartoma, and COVID. This was performed to investigate whether the discovered metabolite markers were specific to lung cancer alone rather than lung disease. More information about the control groups is summarized in Table S1.

### 4.3. Chemicals, Reagents, and Materials Used for the Quantitative Metabolomic Assays

Pure reference standard compounds used for the quantitative metabolomics analysis, Optima™ LC-MS-grade ammonium acetate, phenylisothiocyanate (PITC), 3-nitrophenylhydrazine (3-NPH), 1-ethyl-3-(3-dimethylaminopropyl) carbodiimide (EDC), HPLC-grade pyridine, HPLC-grade methanol, HPLC-grade ethanol, and HPLC-grade acetonitrile (ACN) were purchased from Sigma-Aldrich (Oakville, ON, Canada). Optima™ LC-MS-grade formic acid and HPLC-grade water were purchased from Fisher Scientific (Ottawa, ON, Canada). ^2^H-, ^13^C-, and ^15^N-labeled compounds were purchased from Cambridge Isotope Laboratories, Inc. (Tewksbury, MA, USA) and Sigma-Aldrich (Oakville, ON, Canada). 3-(3-hydroxyphenyl)-3-hydroxypropionic acid (HPHPA) and ^13^C-labeled HPHPA were synthesized in-house as described previously [37]. Multiscreen “solvinert” filter plates (hydrophobic, PTFE, 0.45 μm, clear, non-sterile) and Nunc^®^ 96 DeepWell™ plates were purchased from Sigma-Aldrich (Oakville, ON, Canada).

### 4.4. Stock Solutions, Internal Standard (ISTD) Mixture, and Calibration Curve Standards for Metabolomic Assays

All chemicals, including isotope-labeled compounds, used in this study were weighed individually on a Sartorius CPA225D semimicro electronic balance (Mississauga, ON, Canada) with a precision of 0.0001 g. Stock solutions with proper concentrations for each analyte, and stock solutions of internal standards (ISTD) were prepared by dissolving the accurately weighed chemicals in proper solvents. Calibration curve standards, quality control standards, and working ISTD solutions were prepared by mixing and diluting corresponding stock solutions with appropriate solvents. All standard and ISTD solutions were aliquoted and stored at −80 °C until further use.

### 4.5. Sample Preparation and Liquid Chromatography/Direct Injection Mass Spectrometry for Metabolomic Assays

The plasma samples were analyzed using the same targeted, quantitative MS-based metabolomics approach as in our previous lung cancer biomarker study (14). Mass spectrometric analysis of the diluted plasma extracts was performed on a Qtrap^®^ 4000 tandem mass spectrometry instrument (Applied Biosystems/MDS Analytical Technologies, Foster City, CA, USA) equipped with an Agilent 1260 HPLC (Agilent Technologies, Santa Clara, CA, USA). This LC-MS assay enables the targeted identification and quantification of up to 138 different endogenous metabolites, including amino acids, acylcarnitines, biogenic amines and derivatives, organic acids, uremic toxins, glycerophospholipids, sphingolipids, and sugars. The method employs chemical derivatization (via 3-NPH for organic acids or PITC for amine-containing compounds), analyte extraction, analyte separation, and selective mass-spectrometric detection using multiple reaction-monitoring (MRM) pairs for metabolite identification and quantification. Isotope-labeled ISTDs, along with other ISTDs, are used for accurate metabolite quantification. Details of the method, derivatization strategy, separation protocol, MS methods, calibration, and metabolite quantification process are described in [38].

### 4.6. Data Analysis

Numeric clinical variables were analyzed using a Student’s *t*-test or Mann–Whitney rank sum test, depending on their normality. The normality of the numerical clinical variables was analyzed using a Shapiro–Wilk test. Categorical clinical variables were analyzed by χ^2^ tests. All these tests mentioned above were performed with a *p*-value threshold at 0.05 using the R statistical programming language (R 4.2.1) [39]. Recommended statistical procedures for standard quantitative metabolomic analysis were followed [40]. Metabolites with >80% missing values across all groups were removed from further analysis. For metabolites with less than 80% missing concentrations, values were imputed by using 1/5 of the minimum detectable concentration value for that metabolite. The raw concentrations underwent median normalization, log transformation, and then auto-scaling (mean-centered and divided by the standard deviation of each variable) before further data analysis. Non-parametric univariate analysis and partial least squares discriminant analysis (PLS-DA) were performed by using MetaboAnalyst 5.0 [41]. A 1000-fold permutation test was conducted to determine the likelihood that the observed separation of the PLS-DA was not due to chance. Diagnostic models for lung cancer were developed using logistic regression, incorporating both metabolite and clinical variables. Logistic regression was performed by using R 4.2.1 [39]. Optimal regression models were first identified using the discovery cohort. These models were then confirmed using the validation cohort. The area under the receiver-operator characteristic (AUROC) curves, sensitivities/specificities at selected cut-off points, and the 95% confidence intervals were calculated for both the discovery and the validation sets for all models using the pROC R package [42]. Cut-off points were determined by calculating the Youden Index (J = max {Sensitivity + Specificity − 1}).

## 5. Conclusions

This study validates the use of large-scale, targeted quantitative metabolomics analysis for identifying diagnostic metabolic biomarkers of early-stage NSCLC. Our LR model performed remarkably well in distinguishing stage I and stage II NSCLC patients from the control group, with an AUROC exceeding 90% in both the discovery and validation sets. These results underscore each of the models’ diagnostic strengths and clinical relevance.

These promising results pave the way for the development of a minimally invasive, highly efficient, scalable, and cost-effective lung cancer screening assay. This assay, which would require as little as 10 μL of plasma and which could be processed within minutes using a standard clinical-grade mass spectrometer, represents a practical solution for widespread clinical adoption. Its affordability and ease of use could enhance accessibility to early lung cancer screening, particularly in resource-limited settings.

In a broader context, our study highlights the significant clinical utility of metabolite biomarkers, especially when combined with a patient’s smoking history, for the early detection of NSCLC. This approach offers promising potential to improve patient outcomes through timely intervention, which could lead to increased survival rates and enhanced quality of life. Furthermore, integrating metabolite biomarkers into routine lung cancer screening could offer a more comprehensive diagnostic strategy, reducing false positives and refining treatment decisions. Future research should focus on validating these findings across diverse populations and exploring the integration of metabolite biomarkers with emerging technologies such as deep learning to enhance diagnostic precision. Additionally, expanding this approach to monitor disease progression could unlock new possibilities in personalized medicine. The incorporation of metabolomics into routine cancer screening not only represents a significant advancement in the field of oncology but also has the potential to reshape current healthcare practices.

## Figures and Tables

**Table 1:** Summary of grouping of samples.

**Discovery Set**													
**Class**	**Group**	**Number of Samples**	**Age**			**Histology**		**Gender**		**Smoking Status**			
			**Range**	**Mean**	**Median**	**Adenocarcinoma**	**Squamous Cell Carcinoma**	**Male**	**Female**	**Never**	**Former**	**Current**	Median Pack * × Years (Former + Current)
**Lung Cancer**	**Stage I NSCLC**	275	31–81	64.8	66	200	75	137	138	15	187	73	39
	**Stage II NSCLC**	141	38–82	65.9	66	98	43	68	73	3	110	28	40
	**Stage III + IV NSCLC**	50	49–78	64.8	63	32	18	28	22	7	41	2	35.3
**Control**		214	28–90	60.7	61	/	/	116	98	98	88	28	0.9
**Total**		680	28–90	63.5	64	/	/	349	331	123	426	131	34
**Validation Set**													
**Class**	**Group**	**Number of Samples**	**Age**			**Histology**		**Gender**		**Smoking Status**			
			**Range**	**Mean**	**Median**	**Adenocarcinoma**	**Squamous Cell Carcinoma**	**Male**	**Female**	**Never**	**Former**	**Current**	Median Pack * × Years (Former + Current)
**Lung Cancer**	**Stage I NSCLC**	70	49–79	66.1	67	50	20	26	44	14	40	16	30
	**Stage II NSCLC**	60	59–79	63	63	40	20	20	40	5	50	5	30
	**Stage III + IV NSCLC**	26	42–79	61.7	63	20	6	14	12	0	18	8	40
**Control**		60	49–77	62.6	63	/	/	26	34	38	22	0	0
**Total**		216	42–79	64	65	110	46	86	130	57	130	29	33

* 1 pack = 20 cigarettes.

**Table 2:** The logistic-regression-based optimal model for NSCLC patients vs. controls. Values in square brackets represent measured (unscaled) concentrations of the metabolites. Values in square brackets represent measured (unscaled) concentrations of the metabolites or unscaled amount of smoking (Pack × Year).

	Name of Metabolites	*p*-Value	Odds
**Summary of Each Feature**	Citric acid	7.30 × 10^−7^	0.47
	Tryptophan	1.56 × 10^−4^	0.53
	LysoPC a C18:2	1.44 × 10^−9^	0.31
	Glutamine	1.01 × 10^−5^	2.09
	Succinic acid	1.61 × 10^−2^	0.68
	Citrulline	1.22 × 10^−7^	2.11
	PC aa C38:0	4.79 × 10^−7^	4.48
	PC ae C40:6	4.26 × 10^−6^	0.23
	LysoPC a C20:3	4.79 × 10^−2^	1.40
	Carnitine	9.29 × 10^−2^	1.29
	Amount of Smoking (Pack × Year)	1.56 × 10^−14^	3.55
**Model Performance**	AUC (95% CI)	93.63% (91.76–95.50%)	
	Sensitivity (95% CI)	88.20% (85.19–91.20%)	
	Specificity (95% CI)	85.51% (80.84–90.19%)	
Note: the numeric value of each named metabolite was scaled as follows:			
Citric acid = (log_10_([Citric acid]/102.00) − 1.73)/0.16			
Tryptophan = (log_10_([Tryptophan]/43.10) − 1.34)/0.14			
LysoPC a C18:2 = (log_10_([LysoPC a C18:2]/18.62) − 1.00)/0.19			
Glutamine = (log_10_([Glutamine]/479.00) − 2.40)/0.11			
Succinic acid = (log_10_([Succinic acid]/2.21) + 0.09)/0.07			
Citrulline = (log_10_([Citrulline]/31.30) − 1.22)/0.15			
PC aa C38:0 = (log_10_([PC aa C38:0]/3.63) − 0.29)/0.14			
PC ae C40:6 = (log_10_([PC ae C40:6]/4.38) − 0.37)/0.13			
LysoPC a C20:3 = (log_10_([LysoPC a C20:3]/2.98) − 0.18)/0.15			
Carnitine = (log_10_([Carnitine]/34.64) − 1.26)/0.13			
Amount of Smoking (Pack × Year) = (log_10_([Amount of Smoking (Pack × Year)]/35.00) − 0.92)/0.83			

**Table 3:** The logistic regression-based optimal model for early-stage (Stage I + Stage II) NSCLC patients vs. controls. Values in square brackets represent measured (unscaled) concentrations of the metabolites. Values in square brackets represent measured (unscaled) concentrations of the metabolites or unscaled amount of smoking (Pack × Year).

	Name of Metabolites	*p*-Value	Odds
**Summary of Each Feature**	Citric acid	1.80 × 10^−5^	0.50
	Tryptophan	6.80 × 10^−4^	0.56
	LysoPC a C18:2	6.19 × 10^−10^	0.29
	Glutamine	1.42 × 10^−5^	2.09
	Succinic acid	6.22 × 10^−3^	0.63
	Citrulline	2.03 × 10^−7^	2.12
	PC aa C38:0	7.99 × 10^−8^	5.45
	PC ae C40:6	1.32 × 10^−6^	0.20
	LysoPC a C20:3	3.01 × 10^−2^	1.45
	Carnitine	9.10 × 10^−2^	1.30
	Amount of Smoking (Pack × Year)	1.13 × 10^−14^	4.01
**Model Performance**	AUC (95% CI)	93.74% (91.84–95.64%)	
	Sensitivity (95% CI)	87.98% (84.62–90.86%)	
	Specificity (95% CI)	85.98% (81.31–91.20%)	
Note: the numeric value of each named metabolite was scaled as follows:			
Citric acid = (log_10_([Citric acid]/105.00) − 1.90)/0.53			
Tryptophan = (log_10_([Tryptophan]/42.6) − 1.39)/0.15			
LysoPC a C18:2 = (log_10_([LysoPC a C18:2]/18.43) − 1.04)/0.20			
Glutamine = (log_10_([Glutamine]/472.00) − 2.45)/0.11			
Succinic acid = (log_10_([Succinic acid]/2.23) + 0.08)/0.07			
Citrulline = (log_10_([Citrulline]/30.7) − 1.24)/0.16			
PC aa C38:0 = (log_10_([PC aa C38:0]/3.54) − 0.33)/0.14			
PC ae C40:6 = (log_10_([PC ae C40:6]/4.32) − 0.42)/0.13			
LysoPC a C20:3 = (log_10_([LysoPC a C20:3]/2.94) − 0.23)/0.16			
Carnitine = (log_10_([Carnitine]/34.44) − 1.24)/0.16			
Amount of Smoking (Pack × Year) = (log_10_([Amount of Smoking (Pack × Year)]/33.00) − 0.88)/0.89			

**Table 4:** The logistic-regression-based optimal model for Stage I NSCLC patients vs. controls. Values in square brackets represent measured (unscaled) concentrations of the metabolites. Values in square brackets represent measured (unscaled) concentrations of the metabolites or unscaled amount of smoking (Pack × Year).

	Name of Metabolites	*p*-Value	Odds
**Summary of Each Feature**	Citric acid	1.54 × 10^−4^	0.49
	Tryptophan	2.70 × 10^−4^	0.49
	LysoPC a C18:2	4.21 × 10^−9^	0.27
	Glutamine	7.54 × 10^−5^	2.06
	Succinic acid	2.51 × 10^−2^	0.66
	Citrulline	2.85 × 10^−8^	2.58
	PC aa C38:0	1.19 × 10^−6^	6.30
	PC ae C40:6	7.59 × 10^−6^	0.17
	LysoPC a C20:3	2.42 × 10^−2^	1.52
	Carnitine	4.44 × 10^−2^	1.42
	Amount of Smoking (Pack × Year)	2.67 × 10^−12^	4.43
**Model Performance**	AUC (95% CI)	93.91% (91.86–95.95%)	
	Sensitivity (95% CI)	90.54% (86.91–93.82%)	
	Specificity (95% CI)	85.51% (80.37–90.19%)	
Note: the numeric value of each named metabolite was scaled as follows:			
Citric acid = (log_10_([Citric acid]/112.00) − 1.95)/0.58			
Tryptophan = (log_10_([Tryptophan]/43.1) − 1.40)/0.15			
LysoPC a C18:2 = (log_10_([LysoPC a C18:2]/19.05) − 1.06)/0.20			
Glutamine = (log_10_([Glutamine]/470.00) − 2.44)/0.11			
Succinic acid = (log_10_([Succinic acid]/2.28) + 0.08)/0.07			
Citrulline = (log_10_([Citrulline]/30.6) − 1.24)/0.16			
PC aa C38:0 = (log_10_([PC aa C38:0]/3.53) − 0.33)/0.15			
PC ae C40:6 = (log_10_([PC ae C40:6]/4.34) − 0.42)/0.14			
LysoPC a C20:3 = (log_10_([LysoPC a C20:3]/2.99) − 0.23)/0.16			
Carnitine = (log_10_([Carnitine]/34.74) − 1.32)/0.14			
Amount of Smoking (Pack × Year) = (log_10_([Amount of Smoking (Pack × Year)]/30.00) − 0.76)/0.95			

## Data Availability

The metabolomics data that support the findings of this study are Available online the corresponding author upon reasonable request. The clinical data can be accessed through the IUCPQ upon request using the formal request process.

## Supplementary Materials

The following supporting information can be downloaded at https://www.mdpi.com/article/10.3390/ijms26104519/s1.

## References

[1] Gridelli C., Rossi A., Carbone D.P., Guarize J., Karachaliou N., Mok T., Petrella F., Spaggiari L., Rosell R. (2015). Non-small-cell lung cancer. Nat. Rev. Dis. Primers.

[2] Release Notice Canadian Cancer Statistics: A 2020 Special Report on Lung Cancer. https://www.canada.ca/en/public-health/services/reports-publications/health-promotion-chronic-disease-prevention-canada-research-policy-practice/vol-40-no-10-2020/canadian-cancer-statistics-lung-cancer.html.

[3] Siegel R.L., Miller K.D., Wagle N.S., Jemal A. (2023). Cancer statistics, 2023. CA Cancer J. Clin..

[4] Goldstraw P., Chansky K., Crowley J., Rami-Porta R., Asamura H., Eberhardt W.E., Nicholson A.G., Groome P., Mitchell A., Bolejack V. (2016). The IASLC Lung Cancer Staging Project: Proposals for revision of the TNM stage groupings in the forthcoming (Eighth) edition of the TNM classification for lung cancer. J. Thorac. Oncol..

[5] Saarenheimo J., Eigeliene N., Andersen H., Tiirola M., Jekunen A. (2019). The value of liquid biopsies for guiding therapy decisions in non-small cell lung cancer. Front. Oncol..

[6] Aberle D.R., Adams A.M., Berg C.D., Black W.C., Clapp J.D., Fagerstrom R.M., Gareen I.F., Gatsonis C., Marcus P.M., Sicks J.D. (2011). Reduced lung-cancer mortality with low-dose computed tomographic screening. N. Engl. J. Med..

[7] de Koning H.J., van der Aalst C.M., de Jong P.A., Scholten E.T., Nackaerts K., Heuvelmans M.A., Lammers J.J., Weenink C., Yousaf-Khan U., Horeweg N. (2020). Reduced Lung-Cancer Mortality with Volume CT Screening in a Randomized Trial. N. Engl. J. Med..

[8] Wishart D.S. (2016). Emerging applications of metabolomics in drug discovery and precision medicine. Nat. Rev. Drug Discov..

[9] Qi S.A., Wu Q., Chen Z., Zhang W., Zhou Y., Mao K., Li J., Li Y., Chen J., Huang Y. (2021). High-resolution metabolomic biomarkers for lung cancer diagnosis and prognosis. Sci. Rep..

[10] Derveaux E., Louis E., Vanhove K., Bervoets L., Mesotten L., Thomeer M., Adriaensens P. (2018). Diagnosis of lung cancer: What metabolomics can contribute. Lung Cancer—Strategies for Diagnosis and Treatment.

[11] Yu L., Li K., Zhang X. (2017). Next-generation metabolomics in lung cancer diagnosis, treatment and precision medicine: Mini review. Oncotarget.

[12] Tang Y., Li Z., Lazar L., Fang Z., Tang C., Zhao J. (2019). Metabolomics workflow for lung cancer: Discovery of biomarkers. Clin. Chim. Acta.

[13] Madama D., Martins R., Pires A.S., Botelho M.F., Alves M.G., Abrantes A.M., Cordeiro C.R. (2021). Metabolomic profiling in lung cancer: A systematic review. Metabolites.

[14] Zhang L., Zheng J., Ahmed R., Huang G., Reid J., Mandal R., Maksymuik A., Sitar D.S., Tappia P.S., Ramjiawan B. (2020). A high-performing plasma metabolite panel for early-stage lung cancer detection. Cancers.

[15] Chen Y., Ma Z., Zhong J., Li L., Min L., Xu L., Li H., Zhang J., Wu W., Dai L. (2018). Simultaneous quantification of serum monounsaturated and polyunsaturated phosphatidylcholines as potential biomarkers for diagnosing non-small cell lung cancer. Sci. Rep..

[16] Xu J., Chen Y., Zhang R., Song Y., Cao J., Bi N., Wang J., He J., Bai J., Dong L. (2013). Global and targeted metabolomics of esophageal squamous cell carcinoma discovers potential diagnostic and therapeutic biomarkers. Mol. Cell. Proteom..

[17] Hofmanová J., Slavík J., Ciganek M., Ovesná P., Tylichová Z., Karasová M., Zapletal O., Straková N., Procházková J., Bouchal J. (2021). Complex alterations of fatty acid metabolism and phospholipidome uncovered in isolated colon cancer epithelial cells. Int. J. Mol. Sci..

[18] Chuang S.C., Fanidi A., Ueland P.M., Relton C., Midttun O., Vollset S.E., Gunter M.J., Seckl M.J., Travis R.C., Wareham N. (2014). Circulating biomarkers of tryptophan and the kynurenine pathway and lung cancer risk. Cancer Epidemiol. Biomark. Prev..

[19] Deja S., Porebska I., Kowal A., Zabek A., Barg W., Pawelczyk K., Stanimirova I., Daszykowski M., Korzeniewska A., Jankowska R. (2014). Metabolomics provide new insights on lung cancer staging and discrimination from chronic obstructive pulmonary disease. J. Pharm. Biomed. Anal..

[20] Miyamoto S., Taylor S.L., Barupal D.K., Taguchi A., Wohlgemuth G., Wikoff W.R., Yoneda K.Y., Gandara D.R., Hanash S.M., Kim K. (2015). Systemic metabolomic changes in blood samples of lung cancer patients identified by gas chromatography time-of-flight mass spectrometry. Metabolites.

[21] Ren Y.P., Tang A.G., Zhou Q.X., Xiang Z.Y. (2011). Clinical significance of simultaneous determination of serum tryptophan and tyrosine in patients with lung cancer. J. Clin. Lab. Anal..

[22] Klupczynska A., Dereziński P., Garrett T.J., Rubio V.Y., Dyszkiewicz W., Kasprzyk M., Kokot Z.J. (2017). Study of early stage non-small-cell lung cancer using Orbitrap-based global serum metabolomics. J. Cancer Res. Clin. Oncol..

[23] Rodrigues D., Jerónimo C., Henrique R., Belo L., de Lourdes Bastos M., de Pinho P.G., Carvalho M. (2016). Biomarkers in bladder cancer: A metabolomic approach using in vitro and ex vivo model systems. Int. J. Cancer.

[24] Hur H., Paik M.J., Xuan Y., Nguyen D.T., Ham I.H., Yun J., Cho Y.K., Lee G., Han S.U. (2014). Quantitative measurement of organic acids in tissues from gastric cancer patients indicates increased glucose metabolism in gastric cancer. PLoS ONE.

[25] Fan T.W., Lane A.N., Higashi R.M., Farag M.A., Gao H., Bousamra M., Miller D.M. (2009). Altered regulation of metabolic pathways in human lung cancer discerned by (13)C stable isotope-resolved metabolomics (SIRM). Mol. Cancer.

[26] Tiburcio P.D., Choi H., Huang L.E. (2014). Complex role of HIF in cancer: The known, the unknown, and the unexpected. Hypoxia.

[27] Bamji-Stocke S., van Berkel V., Miller D.M., Frieboes H.B. (2018). A review of metabolism-associated biomarkers in lung cancer diagnosis and treatment. Metabolomics.

[28] Keshet R., Szlosarek P., Carracedo A., Erez A. (2018). Rewiring urea cycle metabolism in cancer to support anabolism. Nat. Rev. Cancer.

[29] You D., Wang D., Wu Y., Chen X., Shao F., Wei Y., Zhang R., Lange T., Ma H., Xu H. (2022). Associations of genetic risk, BMI trajectories, and the risk of non-small cell lung cancer: A population-based cohort study. BMC Med..

[30] Duan P., Hu C., Quan C., Yi X., Zhou W., Yuan M., Yu T., Kourouma A., Yang K. (2015). Body mass index and risk of lung cancer: Systematic review and dose-response meta-analysis. Sci. Rep..

[31] Canadian Cancer Society Risk Factors for Lung Cancer [Internet]. https://cancer.ca/en/cancer-information/cancer-types/lung/risks.

[32] Takiguchi Y., Sekine I., Iwasawa S. (2013). Overdiagnosis in lung cancer screening with low-dose computed tomography. J. Thorac. Oncol..

[33] Jonas D.E., Reuland D.S., Reddy S.M., Nagle M., Clark S.D., Weber R.P., Enyioha C., Malo T.L., Brenner A.T., Armstrong C. (2021). Screening for lung cancer with low-dose computed tomography: Updated evidence report and systematic review for the US Preventive Services Task Force. JAMA.

[34] Wan J.C.M., Massie C., Garcia-Corbacho J., Mouliere F., Brenton J.D., Caldas C., Pacey S., Baird R., Rosenfeld N. (2017). Liquid biopsies come of age: Towards implementation of circulating tumour DNA. Nat. Rev. Cancer.

[35] Mouliere F., Chandrananda D., Piskorz A.M., Moore E.K., Morris J., Ahlborn L.B., Mair R., Goranova T., Marass F., Heider K. (2018). Enhanced detection of circulating tumor DNA by fragment size analysis. Sci. Transl. Med..

[36] Markus H., Chandrananda D., Moore E., Mouliere F., Morris J., Brenton J.D., Smith C.G., Rosenfeld N. (2022). Refined characterization of circulating tumor DNA through biological feature integration. Sci. Rep..

[37] Khaniani Y., Lipfert M., Bhattacharyya D., Perez Pineiro R., Zheng J., Wishart D.S. (2018). A simple and convenient synthesis of unlabeled and ^13^C-labeled 3-(3-hydroxyphenyl)-3-hydroxypropionic acid and its quantification in human urine samples. Metabolites.

[38] Zheng J., Zhang L., Johnson M., Mandal R., Wishart D.S. (2020). Comprehensive targeted metabolomic assay for urine analysis. Anal. Chem..

[39] R Foundation for Statistical Computing (2022). R: A Language and Environment for Statistical Computing.

[40] Wishart D.S. (2010). Chapter 14 Computational Approaches to Metabolomics. Bioinformatics Methods in Clinical Research.

[41] Pang Z., Chong J., Zhou G., de Lima Morais D.A., Chang L., Barrette M., Gauthier C., Jacques P.É., Li S., Xia J. (2021). MetaboAnalyst 5.0: Narrowing the gap between raw spectra and functional insights. Nucleic Acids Res..

[42] Robin X., Turck N., Hainard A., Tiberti N., Lisacek F., Sanchez J.C., Müller M. (2011). pROC: An open-source package for R and S+ to analyze and compare ROC curves. BMC Bioinform..

